# Identification of molecular markers for the early detection of human squamous cell carcinoma of the uterine cervix

**DOI:** 10.1038/sj.bjc.6600038

**Published:** 2002-01-21

**Authors:** Q Cheng, W M Lau, S H Chew, T H Ho, S K Tay, K M Hui

**Affiliations:** Laboratory of Gene Structure & Expression, Division of Cellular and Molecular Research, National Cancer Centre, 11 Hospital Drive, 169610 Singapore; Department of Pathology and Gynaecological Oncology, KK Women's & Children's Hospital, 229899 Singapore; Department of Gynaecological Oncology, KK Women's & Children's Hospital, 229899 Singapore; Department of Obstetrics and Gynaecology, Singapore General Hospital, 169608 Singapore

**Keywords:** human squamous cervical cancer, FIGO stages, gene expression, ribosomal protein S12, molecular diagnostic marker

## Abstract

To identify novel cellular genes that could potentially act as predictive molecular markers for human cervical cancer, we employed RT–PCR differential display, reverse Northern and Northern blot analysis to compare the gene expression profiles between squamous cell carcinoma biopsies and adjacent histo-pathological normal epithelium tissues. Twenty-eight cDNA clones were isolated that were demonstrated to be consistently over-expressed in squamous cell cervical cancer biopsies of FIGO stages 1B to 3B. Most importantly, it was observed that, in addition to their over-expression in cancer lesions, some of these genes are upregulated in the presumably histo-pathological normal adjacent tissues. Of particular interest is clone G30CC that has been identified to be the gene that encodes S12 ribosomal protein. When employed for RNA–RNA *in situ* hybridization experiments, expression of G30CC could be detected in the immature basal epithelial cells of histo-pathological normal tissues collected from cervical cancer patients of early FIGO stages. In comparison, the expression of G30CC was not detected in cervical tissues collected from patients admitted for surgery of non-malignant conditions. These results allow the distinct possibility of employing the ribosomal protein S12 gene as an early molecular diagnostic identifier for the screening of human cervical cancer and a potential target employed for cancer gene therapy trials.

*British Journal of Cancer* (2002) **86**, 274–281. DOI: 10.1038/sj/bjc/0600038 www.bjcancer.com

© 2002 The Cancer Research Campaign

## 

Cancer of the uterine cervix is one of the most frequent malignancies in women worldwide. The 5-year survival rate of recurrent cervical cancer was only approximately 5% mainly due to the absence of effective systemic therapy that would increase the survival of patients with recurrent disease (Burke *et al*, 1987; Piamsomboon *et al*, 1996; Lanciano, 1996). Epidemiological studies demonstrate that the major risk factor for the development of pre-invasive cervical intra-epithelial neoplasia (CIN) or invasive carcinoma of the cervix is related to HPV infection. It has been proposed that infection with certain HPV types (mainly types 16, 18, 31, 33, 35, 39, 45, 51, 52, 56, 58, 59, and 68) are most likely to progress to cancer (Herrero *et al*, 2000). Continuous expression of the HPV oncoproteins E6, E7 and E5 of the high risk HPV subtypes also appears to be necessary for maintaining the malignant phenotype of cervical cancer patients (zur Hausen, 2000). The detection of infection with the known HPV subtypes associated with high risk of cervical cancer has therefore been considered as useful markers for the diagnosis of cervical cancer (Klaes *et al*, 1999). However, the malignant transformation of cervical epithelial cells requires a long latency period. It is estimated that the mean duration of pre-clinical lesions could be up to 16 years (Bos *et al*, 1997). It has been suggested that most of the infections with the known HPV subtypes associated with high risk of cervical cancer usually last less than a year (Ho *et al*, 1998). Moreover, the known HPV subtypes associated with high risk of cervical cancer can also be found in healthy normal woman without clinical evidence of cervical lesions (Kjaer *et al*, 1997). Therefore, determination of the HPV genotypes alone may not be sufficient in assessing the progression of cancer in uterine cervix.

The vast majority of low-grade CIN lesions regress spontaneously, and it has been suggested that approximately 1% of CIN1 lesion and about 10% of CIN3 lesions actually progress to cancer (Duggan *et al*, 1998; Ostor, 1993). Despite the observed low risk for cancer progression, the current treatment of choice for all high-grade CIN cervical lesions is still complete excision of the lesion inclusive of the transformation zone. This is adopted, in the absence of adequate genetic markers to monitor disease progression, to securely prevent the development of invasive carcinomas. Thus, the lack of suitable cancer progression marker results in a significant number of cervical cancer patients being over-treated. Therefore, sensitive and objective diagnostic markers that could assess the potential of the invasiveness of cervical neoplastic cells detected in cervical cancer patients would be of tremendous clinical value. Since it has been estimated that over 75% of all cervical cancer cases reported are squamous cell carcinoma (Vizcaino *et al*, 2000), we have therefore employed differential display as a tool to search for candidate genes with accurate predictive value for squamous cell cervical carcinoma. In the present manuscript, we have focused to study the gene expression profiles of biopsies collected from patients with squamous cell cervical carcinoma of different FIGO stages (FIGO News, 1987) in comparison to the adjacent histo-pathological normal epithelium.

## MATERIALS AND METHODS

### Tissue samples

Surgically resected squamous cell cervical cancer biopsies and the corresponding carcinoma adjacent histo-phenotypical normal epithelium were obtained from 34 patients with informed consent. Punch biopsies with tischler forceps were taken from the tumour lesion for histopathological assessment as well as for RNA analysis. For histopathological analysis, the tissues were fixed in 10% neutral buffered formalin and processed into paraffin blocks. Multiple step sections were made and stained with haematoxylin and eosin. Where indicated, PAS with and without diastase digestion and mucicarmine stains were performed. The tissues collected for molecular analyses were snapped frozen and stored in liquid nitrogen. Normal cervical tissue biopsies collected from four non-cervical cancer patients, with informed consent, were also included in this study. The histology of the presumably normal epithelial tissues collected adjacent to the carcinoma were further ascertained to be normal by pathologists. The staging of the cervical cancer was performed according to the principle recommended by the International Federation of Obstetrics and Gynaecology (FIGO) (FIGO News, 1987).

### Differential display and gene fragments cloning

Total RNA was purified from the cervical cancer biopsies using TRIZOL Reagent (Life Technologies, Grand Island, NY, USA). After treatment with RNase-free DNase I (Promega, Madison, WI, USA), 1 μg of total RNAs were reverse transcribed with the primer P21 (3′-GTTTTTTTTTTTCGAA-5′). The reverse transcribed cDNA were then amplified by PCR in the presence of 1 μCi [α-^33^P]dATP (New England Nuclear, Boston, MA, USA) and either primer P30 (5′-AAGCTTGGTGACA-3′), P31 (5′-AAGCTTAGTCAAG-3′), or P32 (5′-AAGCTTCCACAGC-3′). The PCR cycling parameters were 94°C for 30 s, 40°C for 2 min, and 72°C for 30 s for a total of 40 cycles and followed by one single step at 72°C for 10 min. The PCR products were then separated on Gel-Mix 6 polyacrylamide gel (Life Technologies, Grand Island, NY, USA). Bands that were differentially expressed were excised and the cDNA fragments eluted by boiling the gel pieces in 100 μl H_2_O for 15 min. These cDNA fragments were subsequently employed as templates for PCR re-amplification using similar conditions as described above. Re-amplified cDNA fragments were then cloned into either the pCR2.1 vector via the TA cloning system from Invitrogen (San Diego, CA, USA), or cloned into the pCR-TRAP vector system from GenHunter (Nashville, TN, USA).

### Reverse Northern blot

DNA (2 μg) of the amplified gene fragment of each of the purified clones, along with various amounts of α-actin DNA (0.5, 1, 2, or 4 μg) were blotted on Hybond-N^+^ nylon transfer membrane (Amersham, Piscataway, NJ, USA) using the slot-blot apparatus. The spotted DNA were denatured for 5 min at room temperature with 1.5 M NaCl, 0.5 M NaOH, and neutralized for 5 min at room temperature with 1.5 M NaCl, 1 M Tris-HCl pH 7.4, the membrane was then incubated at 80°C for 2 h to fix the DNA. The^ 32^P-labelled cDNA probes were prepared by reverse transcription of 30 μg of total RNA isolated either from the pooled tumour biopsies of six patients or from their matched normal biopsies using 1000 U of SuperScript™II reverse transcriptase (Life Technologies, Grand Island, NY, USA) in the presence of 60 μCi [α-^32^P]dATP and [α-^32^P]dCTP (NEN, Boston, MA, USA). RNAs were hydrolyzed by incubation for 30 min at 65°C with 9 μl 3 N NaOH in 70 μl-reaction volume. Neutralization of the reaction mixture was performed by the addition of 30 μl 1 M Tris-HCl (pH 7.4), 9 μl 2 N HCl and 22 μl H_2_O. Hybridization was subsequently performed at 40°C. The net fold increase was calculated as the ratio of gene expression obtained with the probe derived from RNA of cancerous tissue over the level of gene expression obtained from the RNA of normal tissues following normalization of the RNA employed with α-actin.

### Northern blot analysis

Twenty micrograms of total RNA were electrophoresed and transferred to Hybond-N^+^ nylon membrane (Amersham, Piscataway, NJ, USA). Cloned gene fragments were labelled using high prime DNA labelling kit (Boehringer Mannheim GmbH, Mannheim, Germany) and employed as hybridization probe. Hybridization and washes were performed essentially as described previously (Soong and Hui, 1992). The signal obtained following hybridization to the house keeping gene G3PDH cDNA probe was employed as an internal reference to standardize the amount of RNA. The signal of hybridization obtained were analyzed and quantitated by densitometric scanning with the BioRad FX PhosphorImager (BioRad, Richmond, CA, USA).

### RNA*–*RNA* in situ* hybridization

The RNA–RNA *in situ* hybridization was performed by DIG (digoxigenin)-labelled cRNA probe using the DIG RNA labelling Kit from Boehringer Mannheim (Boehringer Mannheim GmbH, Mannheim, Germany). Sub-clones with the gene fragment of interest inserted in opposite orientation were selected to synthesize the sense and anti-sense hybridization probe. After linearization of the template DNA at the unique *Bam*HI site, 75 U of T7 RNA polymerase and DIG-UTP were added to synthesis the DIG-labelled transcripts.

Frozen tumour sections (10 μm thick) were sectioned from the frozen tumour biopsies and were mounted directly onto the microscope slides. The sections were fixed in PBS containing 4% paraformaldehyde by firstly incubating at 50°C for 2 min and then for 5 min at room temperature. Pre-hybridization was performed by incubating the sections at 37°C for 1 h in block solution containing 5% skim milk powder, 4× SSPE, 50% deionized formamide, 30 μg herring sperm DNA, 1% SDS and DEPC-treated H_2_O. DIG-labelled cRNA probe (200 ng ml^–1^ was added to the tissue section and incubated overnight at 37°C in hybridization buffer (16.6% dextran sulphate, 5% skim milk powder, 4× SSPE, 50% deionized formamide, 1% SDS and DEPC-treated H_2_O). Following hybridization, the slides were washed twice with 2× SSC at RT for 5 min. The tissue sections were then incubated at RT for 2 h with 100 mM Tris-HCl pH 7.5 and 150 mM NaCl buffer containing 1% BSA and 0.5% alpha-alkaline phosphatase-conjugated anti-DIG antibody (BM, MannLein, Germany). The slides were subsequently developed at RT overnight by soaking in 0.1 M Tris-HCl pH 9.5, 0.1 M NaCl, 0.05 M MgCl_2_, 3.4% NBT, 1.8% BCIP, 2.4% levamisole in the dark. The colour reaction was stopped by transferring the slides to 10 mM Tris-HCl pH 8 and 1 mM EDTA at RT for 5 min and soaked in H_2_O for 5 min at RT. Following counter-staining in Haematoxylin (BDH Laboratory Supplies, Dorset, UK) for 2–5 s, the slides were finally washed with H_2_O at RT and mounted with coverslips in Kaiser's glycerol gelatin solution (Merk KgaA, Darmstadt, Germany). Slides were analyzed using the Olympus BX 60 microscope (Olympus Optical Co. Ltd).

## RESULTS

### Identification of cDNA fragments specifically over-expressed in human cervical cancer biopsies by differential display

Differential display is a PCR-based method that allows the simultaneously comparison of mRNAs from closely related cell populations (Liang and Pardee, 1992; Liang *et al*, 1993, 1994). In this study, we have produced fingerprints of cDNA fragments using mRNAs purified from biopsies of human squamous cell cervical carcinoma and compared these with the corresponding cDNA fingerprints generated from mRNAs of matched histological normal epithelium tissues.

**Figure 1:**
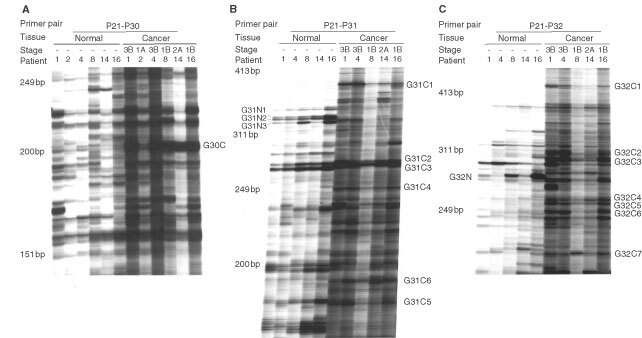
Differential display analysis. Purified total RNA from cervical cancer biopsies was reversed transcribed with the primer P21 (see Materials and Methods). The reverse transcribed cDNA were then amplified by PCR in the presence of 1** **μCi [α-^33^P]dATP and either primer P30, P31, or P32. The PCR products were then separated on polyacrylamide gel. **(A)** RT–PCR differential display with the primer pair P21/P30. **(B)** RT–PCR differential display with the primer pair P21/P31. **(C)** RT–PCR differential display with the primer pair P21/P32. Seventeen bands selected following differential display analyses with the corresponding primer pairs, as indicated in the figure, were employed for subsequent cloning.

**Figure 2:**
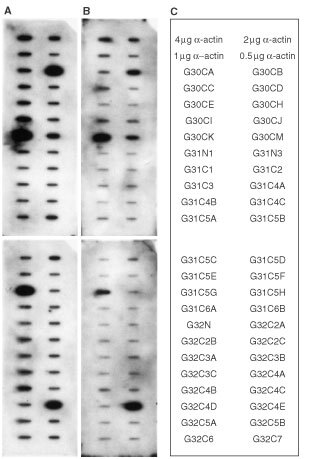
Reverse Northern blot. DNA (2 μg) of the amplified gene fragment of each of the purified clones, along with various amount of α-actin DNA (0.5, 1, 2, or 4 μg) were blotted on Hybond-N^+^ nylon transfer membrane using the slot-blot apparatus. After denaturation, neutralization and DNA fixation, the filters containing the spotted DNA were hybridized to ^32^P-labelled cDNA probes. **(A)** Autoradiogram obtained after Reverse Northern blot hybridization of the spotted cloned gene fragments to a probe prepared by reverse transcription of 30 μg of total RNA purified from the pooled tumour biopsies of six cervical cancer patients. **(B)** Autoradiogram obtained after Reverse Northern blot hybridization of the spotted cloned gene fragments to a probe prepared by reverse transcription of 30 μg of total RNA purified from the corresponding pooled normal tissue biopsies of the same six cervical cancer employed in (**A**). **(C)** The identity and orientation of the cloned gene fragments as spotted on the filter membranes.

One microgram of total RNA extracted from each cervical biopsy was reverse transcribed using the P21 primer. This was followed by a subsequent PCR amplification step employing the P21/P30, P21/P31, or P21/P32 PCR primer pairs. All PCR reactions, including the RT step, were performed in triplicates to ensure reproducibility. In addition, each of the PCR products was analyzed in different electrophoretic separations employing denaturing polyacrylamide gels with various running times. The gel patterns of the amplified PCR products of six independent mRNAs purified from different cancer biopsies and matched normal tissues gave virtually identical gel patterns for each of the PCR primer set employed (Figure 1

). Bands that were differentially expressed were excised and the cDNA fragments purified as described in Materials and Methods. These cDNA fragments were subsequently employed as templates for PCR re-amplification using conditions as described in Materials and Methods. Re-amplified cDNA fragments were then cloned into either pCR2.1 pCR-TRAP. Twenty-eight cDNA clones which level of expression were subsequently confirmed by Reverse Northern analysis to be significantly increased in the cervical cancer biopsies in comparison to their matched histological normal tissues with the net folds of increase ranging from 1.7 to 4.3 (Figure 2

).

Through the BLASTn sequence search (National Centre for Biotechnology Information), most of the clones identified are either homologous to previously reported genes in the Genbank or are known expressed sequence tags (EST) (Table 1

**Table 1:**
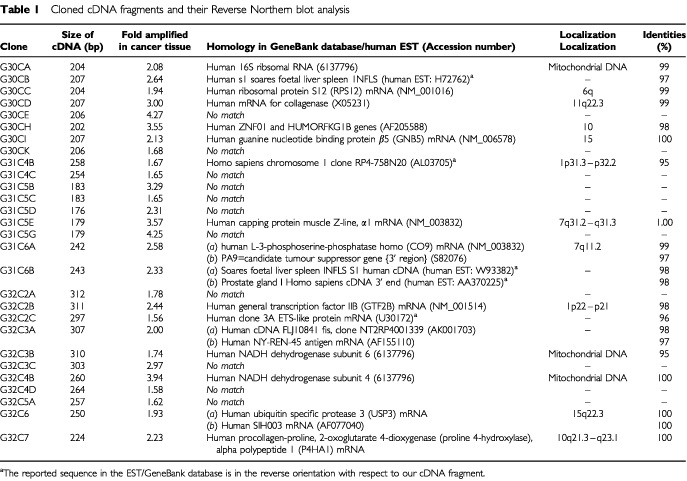
Cloned cDNA fragments and their Reverse Northern blot analysis

). The DNA sequences of clones G30CC, G30CD, G30CI, G30CJ, G31C5H, G32C3B, G32C4B and G32C7 are located within the coding regions of the reported genes, while the DNA sequences of clones G31C4B and G32C2C are in the reverse orientation with respect to that of the reported gene sequences in the Genbank. The DNA sequences for clones G30CB and G31C6B were also found to be in the reverse orientation with reference to the sequences of two reported EST clones. Eleven of the 28 clones, viz. G30CE, G30CK, G31C4C, G31C5B, G31C5C, G31C5D, G31C5G, G32C2A, G32C3C, G32C4D and G32C5A appear to be novel gene sequences as they do not match to any of the reported gene sequence in the GenBank or EST databases (Table 1).

### Expression of the cloned cDNA fragments in various FIGO stages of human cervical cancer

**Figure 3:**
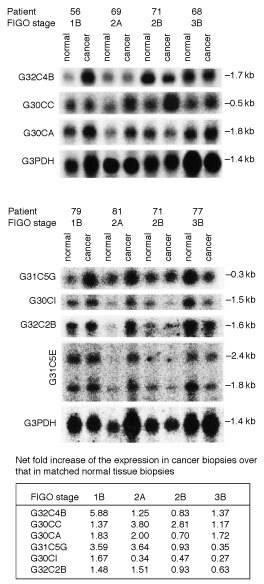
Northern blot analysis employing clones G32C4B, G30CC, G30Ca, G31C5G, G30CI, G32C2B and G31C5E as probes with total RNA obtained from human squamous cell cervical carcinoma biopsies of different FIGO stages and their corresponding adjacent normal tissues. The summary table shows the folds of increase of the cloned cDNA fragments in the various tumour biopsies in comparison to their matched normal counterparts after normalization to G3PDH mRNA.

To determine if the level of gene expression of some of the cloned genes correlate with the tumorigenicity of human squamous cell cervical cancer, Northern blot analyses were performed. mRNA from cervical cancer biopsies of FIGO stages 1B (four patients), 2A (three patients), 2B (two patients), and 3B (four patients), along with their matched normal tissue biopsies were isolated. The level of expression of the cloned genes identified in this study in cervical cancer biopsies of various FIGO stages was compared to that of their matched normal tissues. Our results demonstrated that expression of clone G30CC was consistently upregulated in the cervical cancer tissue biopsies examined (Figure 3

). Cervical cancer biopsies obtained from stage 2A patients gave the highest level of expression of G30CC (Figure 3). DNA sequence homology analyses suggested that clone G30CC is homologous to the human ribosomal protein S12 mRNA (Table 1).

Similarly, DNA sequence homology analyses suggested that clones G30CA and G32C4B are of mitochondrial origin with clone G32C4B encodes the mitochondrial subunit NADH dehydrogenase 4, and clone G30CA is homologous to the human 16S ribosomal RNA that is required for the translation of mitochondrial subunits (Table 1). The expression of these two genes was greatly upregulated when early stages (1B and 2A) squamous cell cervical cancer biopsies were examined (Figure 3). Expression of clone G32C4B was increased by up to six-fold in stage 1B cervical cancer biopsies in comparison to the corresponding adjacent normal tissues (Figure 3).

One of the novel genes identified in this study, clone G31C5G, has been demonstrated to be specifically upregulated in stages 1B and 2A human squamous cervical carcinoma (Figure 3). It was found that the level of expression of both clone G30CI (human guanine nucleotide binding protein β5) and clone G32C2B (human general transcription factor IIB) were greatly upregulated in stage 1B squamous cell cervical cancer. In addition, the expression of clone G32C2B was also upregulated in stage 2A cervical cancer biopsies (Figure 2). Two hybridized bands (2.4 and 1.8 kb) could be observed when clone G31C5E was employed as the hybridization probe in our Northern blot analysis. When DNA sequence homology analyses were performed, the best matched in the homology search to clone G31C5E in the GenBank DNA sequences was the human capping protein muscle Z-line α1 mRNA which is of 2.4 kb. If clone G31C5E is indeed the human capping protein muscle Z-line α1gene, the nature of the 1.8 kb gene product detected in our Northern blot study is presently unknown.

More importantly, through the Northern blot analyses, we demonstrated that the expression of these seven cloned genes identified were also elevated in the matched histological normal tissue biopsies collected together with the different FIGO stages of the squamous cervical cancer biopsies (Figure 3). To explore the potential clinical significance of the upregulation of these genes in the histologically normal cervical tissues adjacent to the tumour proper and to identify the cell types that expressed the gene product in question, one of the clones, clone G30CC, was employed for RNA-RNA *in situ* hybridization study. Both the anti-sense and sense orientations of clone G30CC were synthesized as described in the Materials and Methods and employed as probes for *in situ* hybridization studies. The sense probe was employed as a negative internal control for the *in situ* hybridization.

**Figure 4:**
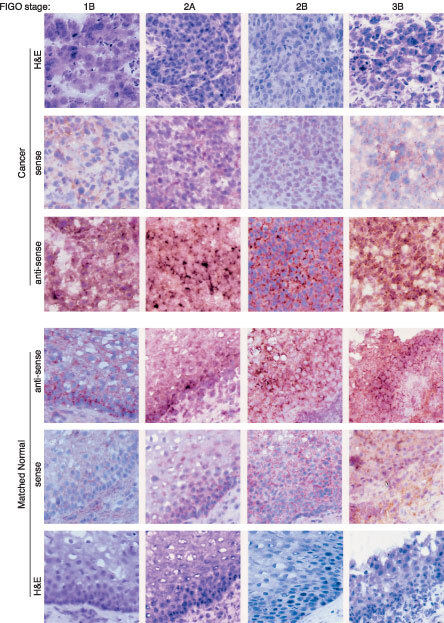
Expression of G30CC in human squamous cell cervical carcinoma of different FIGO stages (1B, 2A, 2B and 3B), as well as their corresponding adjacent normal via RNA–RNA *in situ* hybridization analysis. The RNA–RNA *in situ* hybridization was performed by DIG-labelled cRNA probe using the DIG RNA labelling Kit from Boehringer Mannheim. Sub-clones with the G30CC gene fragment inserted in opposite orientation were selected for the synthesis of the sense and anti-sense hybridization probes. Frozen tumour sections (10 μm thick) were sectioned from the frozen tumour biopsies and reacted with the DIG-labelled cRNA probe. After hybridization and washing, the slides were analyzed using the Olympus BX 60 microscope. The histology identifications were performed on consecutive tissue sections following H&E staining. The magnification shown was 600×.

*In situ* hybridization studies were performed using frozen sections of squamous cell cervical cancer biopsies of various FIGO stages and their matched histological normal tissues. A total of 14 patients of different FIGO stages were analyzed by *in situ* hybridization and the results of a representative patient were shown (Figure 4

). The histology identifications were performed on consecutive tissue sections following H&E staining. When the normal tissue sections were examined, the basement membrane is intact and the immature epithelial cells were of uniform size and are confined to the basal-layer (Figure 4). The mature cells were normally found in the para-layers (Figure 4). On the other hand, in the case of carcinomas, cellular and nuclear variations of different sizes and shapes could be observed (Figure 4).

**Figure 5:**
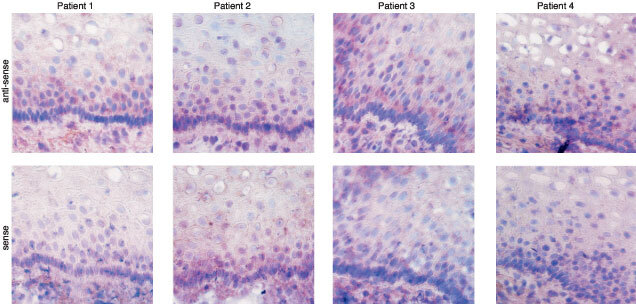
Expression of G30CC in normal epithelium of tissue biopsies obtained from four different non-cervical cancer patients via RNA–RNA *in situ* hybridization analysis. Sub-clones with the G30CC gene fragment inserted in opposite orientation were selected for the synthesis of the sense and anti-sense hybridization probes. The magnification shown was 600×.

For *in situ* hybridization studies with clone G30CC as probe, positive signals were mainly observed in the cytoplasm (Figure 4). Expression of clone G30CC was consistently detected in cervical carcinomas of FIGO stages 1B, 2A, 2B and 3B (Figure 4). Although the G30CC anti-sense probe did not hybridize to the para-epithelial cell layers of matched normal tissues obtained from FIGO stages 1B and 2A cervical cancer patients, specific hybridization could be detected within the immature basal epithelial cell layers (Figure 4). More significantly, when the matched normal tissues obtained from FIGO stages 2B and 3B cervical cancer patients were studied with the G30CC anti-sense probes, strong hybridization signals could be obtained in the immature epithelial cells of histological normal tissue sections (Figure 4). Conversely, G30CC expression could not be detected when normal tissue sections collected from non-cervical cancer patients were tested (Figure 5

).

## DISCUSSION

To identify tumour-specific molecular alterations in human squamous cervical cancer, we have employed differential display and cDNA gene cloning to compare the global gene expression profiles of six tissue biopsies of cervical cancer patients of different FIGO stages with their corresponding matched normal tissue biopsies (Figure 1). We had chosen to utilize biopsies obtained from cervical cancer patients of different FIGO for our differential display studies. This is to avoid the generation of bias against isolating ‘false positive’ differentially displayed cDNAs fragments from a specific type of tumour biopsy (Liang *et al*, 1993). We reasoned that true differential displayed bands that are important for carcinogenesis should be generically up-regulated or down-regulated in most, if not all, cervical cancer samples when compared to the adjacent normal tissues and that these bands should be reproducibly generated by the differential display analyses. A total of 44 cDNA clones were initially isolated from the 17 distinct bands obtained following differential display analyses with the three sets of primers employed. We could isolate more than one cDNA clone from a single distinctive differential display band. This is most likely to be attributed to the co-migration of gene fragments during electrophoretic separation. All the clones purified have been verified to have the expected PCR primer sequences incorporated at their 5′ and 3′ ends by DNA sequencing. The expression of 28 of these 44 cDNA clones were subsequently confirmed by reverse Northern blot analysis to be upregulated in human squamous cell cervical cancer biopsies (Figure 2 and Table 1).

Interestingly, results of our Northern blot analyses indicated that the levels of expression were upregulated for several different categories of genes in the corresponding adjacent histopathological normal tissue biopsies collected from patients with advanced stage cervical cancer (Figure 3). These include genes for energy consumption (NADH dehydrogenase 4, G32C4B), translation (ribosomal protein S12, G30CC; 16S ribosomal RNA, G30CA), transcriptional regulation (general transcription factor, G32C2B), signal transduction (guanine nucleotide binding protein β5, G30CI), and others (G31C5G and G31C5E (Figure 3). It is likely that as the carcinoma spread, the external adjacent, presumably still histological normal, tissues are undergoing molecular changes that are reflected in the observed corresponding alterations in their gene expression.

The strategy adopted in this study to isolate tumour-specific genes has certain limitations. Throughout our study, relatively large tissue biopsies were utilized. These tissues intrinsically represent a vast mixture of cell types (i.e. stroma, neovascularized endothelium, epithelial nest and inflammatory cells). It would be impossible to rule out some of these biological differences in the tumour biopsies to that of their normal counterpart. In addition, there may be inherent differences between the adjacent squamous epithelia and squamous epithelia within the transformation zone. Although every precaution had been taken to collect the corresponding normal tissues, in some cases, there might be traces of contaminating tumour tissues especially in some of the advanced cervical cancer cases studied. The results reported in this study, therefore, may most likely to reflect the importance of the cervical microenvironment. To clarify these issues, we have performed RNA-RNA *in situ* hybridization using G30CC as a representative hybridization probe.

Results of our RNA-RNA *in situ* hybridization analyses indicated that G30CC was consistently over-expressed in cervical cancer biopsies collected from patients of FIGO stages 1B to 3B diseases (Figure 4). It was also observed that the expression of clone G30CC was significantly increased in the corresponding adjacent histological normal biopsies obtained from cervical cancer patients having stage 2B and 3B diseases (Figure 4). The expression of G30CC was also upregulated within the immature epithelial cells in tissue sections of biopsies collected from early stage (1B and 2A) cervical cancer patients (Figure 4). It is therefore most likely that the expression of G30CC has been molecularly modified in the phenotypically normal cervical epithelium tissues adjacent to carcinoma.

DNA sequence homology analyses showed that clone G30CC is homologous to the human ribosomal protein S12 mRNA (Table 1). The over-expression of certain ribosomal protein mRNA has been reported to correlate with tumorigenicity (Chanseob *et al*, 1998; Henry *et al*, 1993; Kumar *et al*, 1999). The function of certain ribosomal proteins has also been implicated in the regulatory processes that may be important in carcinogenesis (Erikson and Maller, 1989; Ziemiecki *et al*, 1990). The over-expression of ribosomal protein has also been suggested to be an early event in the neoplastic progression to malignancy (Pogue-Geile *et al*, 1991). The expression of ribosomal protein S12 gene had been reported to increase in adenomatous polyps when compared to normal mucosa (Pogue-Geile *et al*, 1991). These observations are consistent with our results that the expression of G30CC was upregulated for cervical cancer biopsies collected from patients of early FIGO stages (Figure 3B) and suggests the possibly concomitant increase expression of clone G30CC with the onset of cervical neoplasia.

Events such as genetics mutations and alterations in the regulation of signal transduction pathways play important roles in the initiation of cancer (Hartwell and Kastan, 1994; Weinberg, 1996). During the progression of cancer, the local microenvironment has been demonstrated to contribute significantly for the growth of tumour (Tlsty and Hein, 2001). The local microenvironment can influence the growth of tumours via the continuous enhancement of proliferation through a paracrine mechanism (Tlsty and Hein, 2001). Neoplastic epithelial cell could produce mitogenic factors such as IGF (insulin-like growth factor) and KGF (keratinocyte growth factor) to activate fibroblasts (Basset *et al*, 1990; Chiquet-Ehrismann *et al*, 1986; Ronnov-Jessen *et al*, 1996; Singer *et al*, 1995; Wright *et al*, 1994). These factors may in turn promote the proliferation of neoplastic epithelial cells either by the modulation of extracellular matrix or the release of bound growth factors (Basset *et al*, 1990; Chiquet-Ehrismann *et al*, 1986; Ronnov-Jessen *et al*, 1996; Singer *et al*, 1995; Wright *et al*, 1994). In this context, purified carcinoma-associated fibroblast cells have been shown to exhibit an alteration in the expression of growth factors (Ellis *et al*, 1994; Frazier and Grotendorst, 1997; Nakamura *et al*, 1997; Yan *et al*, 1993; Yee *et al*, 1991). Changes in stromal cells have also been postulated to enhance the tumorigenic phenotypes of epithelial cells (Olumi *et al*, 1999; Rinehart and Torti, 1997; Skobe and Fusenig, 1998). This activated stromal microenvironment may affect the profile of gene expression of the adjacent epithelial cells. This hypothesis is supported by our current results demonstrated that certain genes, such as the ribosomal protein S12 gene, is upregulated in the adjacent tissues of progressive cancers.

The presence of integrated HPV DNA is detected in over 90% of cervical carcinoma (Bosch *et al*, 1995). Moreover, HPV16 DNA, a high-risk HPV subtype for cervical cancer, can be found in more than half of cervical cancers (Ronco *et al*, 1998). Integration of the HPV genome, in most cases, results in the expression of the viral oncogenes E6 and E7 and the co-transcribed cellular sequences (Klaes *et al*, 1999). It was clearly demonstrated that the expression of these oncogenes is essential for the induction and maintenance of the neoplastic phenotype of cervical cancer cells (zur Hausen, 2000). Conceivably, the activated microenvironment induced by the adjacent cervical cancer cells might facilitate the expression of the integrated HPV viral oncogenes and enable the existing phenotypical normal cells to be more prone for clonal outgrowth into cancer cells. The next obvious objective is to evaluate the possible role of the ribosomal protein S12 gene in pre-invasive cervical cancer.
